# Continuous and Long-Term Measurement of Reticuloruminal pH in Grazing Dairy Cows by an Indwelling and Wireless Data Transmitting Unit

**DOI:** 10.1155/2012/236956

**Published:** 2012-11-05

**Authors:** J. Gasteiner, T. Guggenberger, J. Häusler, A. Steinwidder

**Affiliations:** Federal Agricultural Research and Education Centre Raumberg-Gumpenstein, Institute of Farm Animal Welfare and Farm Animal Health, A-8952 Irdning, Austria

## Abstract

The aim of the present study was the continuous measurement of ruminal pH in grazing dairy cows to monitor the diets effects on ruminal pH value. A novel indwelling pH-measurement and data transmitting system was given to 6 multiparous cows orally. Ruminal pH was measured every 600 sec over a 40 d period. After barn feeding and changeover to pasture, the following 3 treatments (2 cows/treatment) were included in the measurement period: continuous grazing (G), continuous grazing plus 4 kg/d of hay fed twice daily (GH), and continuous grazing plus 4 kg/d of concentrate (GC). Ruminal pH decreased significantly (*P* < 0.05) from 6.58 ± 0.15 to pH 6.19 ± 0.19 during feed changeover to pasture. Mean ruminal pH for G, GH, and GC was 6.36, 6.56, and 6.01. Mean 24-h minimum pH was 5.95, 6.20 and, 5.58. The time pH was below 6.3, 6.0, 5.8, and 5.5, for G it was 583, 91, 26, and 3 min/d, for GH it was 97, 12, 0, and 0 min/d and for GC it was 1126, 621, 347, and 101 min/d, respectively. Results were significantly influenced by the diet. The indwelling pH-measurement and data transmitting system is a very useful and proper tool for long-term measurement of ruminal pH in cows.

## 1. Introduction

Rumen acidosis, mainly occurring as subacute rumen acidosis (SARA), is characterized by abnormally low rumen pH. SARA is a widely spread problem in high yielding dairy cows [1] but also in grazing dairy cattle [2, 3]. The risk of SARA is increased in production systems feeding rations with high sugar and starch contents. In these cases, we can find substitution of rations components containing higher amounts of physically effective fibre, and thus, rumination and production of neutralizing saliva are reduced [4–6]. In Irish dairy cows a frequency of 11% for rumen acidosis (pH ≤ 5.5) is indicated, and 42% of examined cows were marginally affected (pH ≤ 5.6–5.8) [3]. In Australian dairy cows, predominantly fed on pasture and concentrates, a frequency of 10.2% for SARA (pH 5.74 ± 0.47) was found [2]. 

The negative effects of SARA on animal health in dairy cows are reduced; DMI, decreasing body condition, diarrhoea, ruminitis and inflammation, caudal vena cava syndrome, displacement/ulceration of the abomasum, laminitis and immunosuppressive disorders [5, 12–14]. 

SARA is difficult to diagnose in the field [1]. The examination of rumen fluid is the most meaningful criterion to evaluate the fermentation conditions, and determination of reticuloruminal pH is the definitive test for SARA [15–17]. 

Reticuloruminal pH in grazing cattle can be measured in rumen fluid, which is either collected by a stomach tube [2, 18, 19] or by rumenocentesis [2, 3, 20]. The technique used to collect ruminal fluid affects the outcome of measured pH values [7, 21, 22]. Strabel et al. [22] found that samples taken by stomach tube showed values, that were 0.5 pH-units higher than those taken by rumenocentesis because samples were contaminated by saliva. Rumenocentesis might have negative effects on animal health [22]. 

Recent techniques use indwelling pH probes placed in the rumen [1] or in the reticulum [23, 24]. Continuous monitoring of reticuloruminal pH is advantageous due to the possibility of recording diurnal [1, 25, 26]. Techniques for continuous measurement of ruminal pH were used for a series of scientific investigations [16, 23, 25, 27, 28]. In order to achieve the collected data, the memory chip has either to be removed via rumen [25, 27–31] or the data are transmitted by cable to an external unit, which is fixed onto the animal [16]. 

 Gasteiner et al. [26] described and evaluated a method for measuring reticuloruminal pH continuously, which uses a wireless data transmitting unit allowing long-term investigations. Rumen cannulated cattle are not essential when using this technique as the probes are given orally.

The aim of the present study was to continuously measure reticuloruminal pH in 6 grazing dairy cows over a 40 d period continuously. During this period, cows had a feed changeover from barn feeding to pasture. While pasturing, animals were fed concentrates and hay in differing quantities, and the diets influence on reticloruminal pH was monitored.

## 2. Material and Methods

The study was conducted at the Federal Agricultural Research and Education Centre Raumberg-Gumpenstein (HBLFA) in the province of Styria (Austria) during May and June, 2010.

### 2.1. Technical System

For monitoring reticuloruminal pH, an indwelling and wireless data transmitting system (*smaXtec animal care GmbH, Graz, Austria*) was used [26]. The measurement interval was 600 seconds, and stored data were transmitted using the ISM-Band (433 MHz). The system was controlled by a microprocessor. Data (pH, temperature) were collected by means of an analogue to digital converter (A/D converter) and stored in an external memory chip. Due to its dimensions (length: 12 cm; width: 3.5 cm; weight: 210 g), this indwelling system can orally be administered to an adult cow, and it is break-proof and resistant to rumen fluid [32]. 

Calibration of the pH-probes was performed using pH 4 and pH 7 buffer solutions at the beginning of the experiment.

### 2.2. Feeding Trial

Grazing period started at the beginning of April, and a gradual transition was done from barn to pasture feeding. During the grazing period, cows had free access to a continuously grazed sward, and sward height was estimated with *“Filip's Folding Plate Pasture Meter.”*

The trial was performed using 6 lactating Austrian Holstein Friesian dairy cows (milk yield 21.4 kg/d ±6.8 kg; parity 3.8 ± 2.2; DIM 193 ± 118; BW 556 kg ±34). During the first period of investigation (from day 1 to day 26), all 6 cows had the same treatment: after a 2-day barn-feeding period (phase 1), whilst the 6 cows had been fed grass silage (50%), hay (30%), and concentrate (20%), animals were given pasture 10 hours/d for 5 days (phase 2). Additional feeding during this phases was grass silage, hay and grain-based concentrate. From day 7 to day 26 the cows spent 20 hours/d on pasture (continuous grazing), only twice daily interrupted for 2 hours for milking and for additional feeding. Feed intake (kg DM/d) is shown in Table 1.

Beginning, from day 27, 3 treatments (2 cows per treatment) were conducted; continuous grazing and 1.2 kg hay/d additional feeding (G), continuous grazing plus 4.0 kg/d of grain-based concentrate offered in two equal rations during milking time plus 1.2 kg hay/d additional feeding (GC), and continuous grazing plus 3.3 kg/d of hay (GH), also fed twice daily. Treatment diets were fed individually as the cows were tied during these feedings. So each individual cow was an experimental unit for that part of the study. 

Hay (7 orts; 5.4 MJ NEL/kg DM; 535.8 g NDF/kg DM) and grass silage (3 orts; 5.7 MJ NEL/kg DM; 494.1 g NDF/kg DM) were harvested and cut (8 cm) during the stage of heading. Grain-based concentrate (7 orts; 7.7 MJ NEL/kg DM; 199.3 g NDF/kg DM) was ground. Individual feed intake during barn feeding was measured twice daily by weighing offered feedstuff (separate feeding and weighing of single components) and by subtracting the remained rest of the feedstuff. Hay and grass silage were single cutting, and representative grab samples were taken weekly near to the time of feeding for chemical analysis of nutrient contents. To sample grass from pasture, a part of the plot was fenced out from grazing. During experimental period the fenced area was harvested three times, and grass samples were analysed chemically. The nutrient content of grass changed during the investigation (3 orts; 6.9–6.7 MJ NEL/kg DM; 354.3–384.2 g NDF/kg DM).

The chemical analyses (Weende crude nutrients, cell wall analyses) of hay, grass silage, concentrate, and pasture were carried out after dry matter determination by conventional methods [33–35]. 

The total DM intake had been calculated using the feed intake prediction equation of Gruber et al. [36]. In this equation both, nutritional factors (forage quality and composition, concentrate level) and animal factors (milk yield, live weight, stage of lactation, and breed) are used as predictors for feed intake. On the basis of total DMI, the individual nutrient intake was calculated by multiplication with concentration of nutrient contents. Pasture intake was estimated as the difference of calculated total DMI and the weighed DMI of hay, grass silage and grain. 

### 2.3. Statistical Analysis

Statistical analysis was performed by GLM (ANOVA, Statgraphic Plus 5.1) followed by Dunnett's multiple comparison method to determine the differences mean pH of different feeding strategies. *P* values less than 0.05 were considered being significant.

## 3. Results

**Figure 1:**
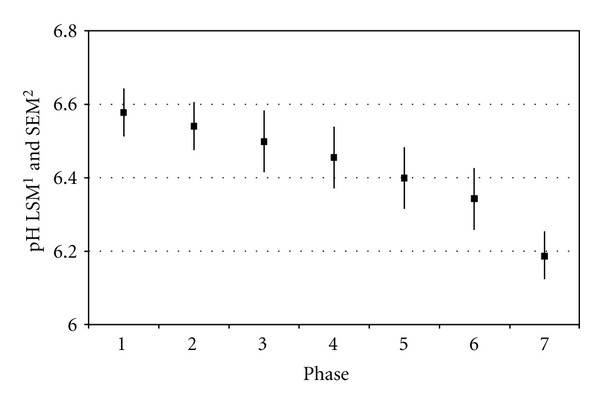
Phase patterns of mean reticuloruminal pH during the first 26 days of examination (feed changeover period) from barn feeding (phase 1), 10 h/d-pasture (phase 2), and 20 h/d (phases 3–7). Vertical bars indicate SE of phase means. ^1^Least squares means, ^2^SE of least squares means.

The period of examination was 40 days and all indwelling probes worked thoroughly so that 144 datasets/cow/day for the reticuloruminal pH could be recorded. The antennas to pick up the radio signal were installed in the milking parlour, and data were read out twice daily during milking. During phases 1–7 (days 1–26) mean reticuloruminal pH significantly (*P* < 0.05) decreased in all 6 cows. Mean reticuloruminal pH during phase 1 was 6.58 ± 0.15 and pH steadily decreased to pH 6.19 ± 0.19 during phase 7 (Figure 1). 

The 24-h minimum pH constantly decreased from phase 1 (6.23) to phase 7 (5.75), and the time pH was below 5.8 significantly (*P* < 0.05) increased during phase 7 (96 min/d) when compared to phase 1 and phase 2 (0 min/d).

**Figure 2:**
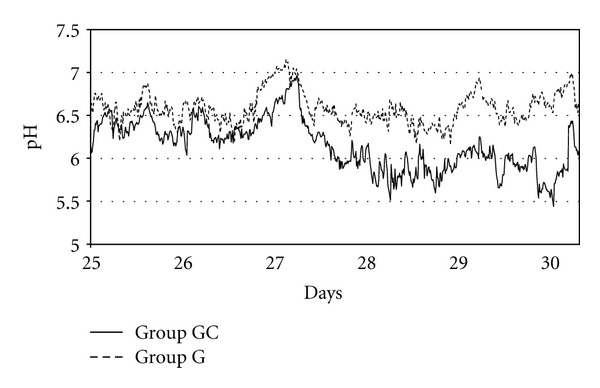
Diurnal patterns of mean reticuloruminal pH of cows from group G and GC (20 hours grazing per day) between day 25 and day 30 with the diet change of group GC to additional concentrate feeding (GC) on day 27, whilst group G was still receiving only pasture (20 h/d). Group GH is not shown in this figure due to clear arrangement.

Up to day 26, no significant differences in reticuloruminal pH could be seen between the 6 cows while only grazing 20 hours per day. After that, cows were “off feed” for 6 hours on day 27 (Figure 2), which led to a distinct elevation of the reticuloruminal pH. Immediately after feeding, reticuloruminal pH decreased in both groups. Whilst pH-decrease in group G remained at the level of day 26, reticuloruminal pH of group GC declined to a more acidotic stage.

It could be shown that additional feeding of 4 kg concentrate significantly (*P* < 0.05) reduced reticuloruminal pH (GC) (Table 2). 

Mean reticuloruminal pH on GC diet (6.01) was significantly (*P* < 0.05) reduced, whilst reticuloruminal pH in G did not change when compared to the course prior to the “off feeding” period. Mean 24-h-minimum pH in GC was 5.58. The time pH was below 5.8 when for GC it was 347 min, and when was below 5.5 it was 101 minutes. However, in group G reticuloruminal pH was below 5.8 for 26 min/d and for GH it was 0 min/d. Reticuloruminal pH in GH was highest (6.56 ± 0.15). 

## 4. Discussion

The present study has demonstrated that grazing dairy cows are potentially at risk of developing SARA, especially during the feed changeover period from barn feeding to pasture and when grain-based concentrates are fed additionally while pasturing. 

Repeated measurement of reticuloruminal pH remains the only way to quantify the balance between acid production, acid removal, and buffering capacity in the rumen [37]. In our study pH-value was measured at the bottom of the reticulum continuously, as the probes, orally given, will end up there due to their weight at least 24 hours after administration [26, 38]. It was also proven that the measurement of the reticular pH in cattle is representative for the ruminal pH [38], for this very reason the term “reticuloruminal pH” is used in our work. Gasteiner et al. [26] evaluated the indwelling system by a comparison of measuring standardized pH dilutions (pH 4, pH 7) prior and after in vivo measurements; coefficient of correlation in this study was 0.9987. Drift pH 4 was 0,197±0,070, and drift pH 7 was 0,107±0,088.

It is well recognized that cattle require an adaptation period of at least 7 d to make the transition from different diets without any complications. Clinical and subclinical acidosis can experimentally be induced by eliminating this adaptation phase or by abruptly changing diet compositions [39, 40]. In our study, all 6 cows were treated the same way over the time of feed changeover until day 27. During these phases mean reticuloruminal pH decreased in all 6 cows significantly (*P* < 0.05). Whilst mean reticuloruminal pH during phase 1 was 6.58 ± 0.15, pH decreased to pH 6.19 ± 0.19 during phase 7, but when having a look at 24-h minimum pH it was 6.23 and 5.75 respectively. Cows of having a rumen pH of <5.8 were characterized to be affected from SARA [20].

Mean reticuloruminal pH of grazing cows in the present study was 6.36 ± 0.16, and results of the feeding trials were significantly influenced by the diet (*P* < 0.05). Mean reticuloruminal pH for G, GH, and GC was 6.36, 6.56, and 6.01. In a review of 20 studies, in which fresh pasture made up >70% of the diet, daily mean pH was reported to be 6.16 (range 5.6–6.7) [41]. These findings are within the range of our results. Decrease of reticuloruminal pH can be seen as a consequence of changing pasture nutrient components. In our trial NDF in phases 1–7 was decreasing from 454 g/kg DM during phase 1 to 391 g/kg DM during phase 7, whilst energy increased from 5.99 MJ NEL to 6.67 MJ NEL. Where the assessment of feeds, individual cows, and the herd suggests suboptimal ruminal pH, the provision of a high NDF supplementary feed source, such as hay, may benefit the health and productivity of cows [4].

24-h minimum pH for G, GH and GC, in our study were 5.95, 6.20, and 5.58, which significantly differed between feeding groups (*P* < 0.05). Animals in GC were affected by SARA according to the definition of Nordlund and Garrett [20].

Daily mean of reticuloruminal pH may not adequately represent its highly variable characteristics [41]. While daily mean reticuloruminal pH could remain relatively high, the deepest pH value per day (nadir) of the reticulorumen might be <5.8 and be considered acidotic. Such specific analysis and calculation will only be possible if using a continuous measurement system. In the present study, 144 pH measurements/cow/day were carried out over a period of 40 days to receive a representative amount of data. The time pH was below 6.3, 6.0, 5.8, and 5.5, for G it was 583, 91, 26, and 3 min/d, for GH it was 97, 12, 0, and 0 min/d, and for GC, time was 1126, 621, 347, and 101 min/d, respectively. Dietary NDF may be an explanation for this result. NDF was highest in G (416 g/kg DM), and it was lowest in GC (359 g/kg DM). It can be stated that highly fermentable diets require adequate amounts of NDF/fiber to reduce the risk of subacute and clinical acidosis. 

Different groups of authors built segments over the time/value relationship of the measured pH and calculated the time above or below a certain defined pH threshold. Nocek et al. [30] showed the effect of increasing amounts of the percentage of grain in the diet on mean reticuloruminal pH within specific ranges (<5; >5, <5.5; >5.5, <6). Plaizier et al. [6] showed the duration of the pH value below 5.6 in minutes per 24 hours. Segmentation over time/value eases interpretation. Penner and Oba [42] showed that duration (h/d) and area (pH × min/d) were not affected by increased dietary sugar concentration. 

It could be demonstrated in our study that when 4 kg/d grain-based concentrate was fed to grazing cows additionally, reticuloruminal pH decreased, significantly. These findings are in accordance with a trial that found that a reduced time is spent during grazing period and a lower feed intakes are needed when supplementing 6 kg grain-based concentrate, fed 2 times daily or 4 times daily [43]. Due to the progressive changing of the pastures nutrient components, mainly fibre, protein, and sugar, no Latin square design was carried out in our study. Good quality pastures combined with grain-based concentrate are highly fermentable diets and when digested we find high concentrations of VFA and relatively low ruminal pH [44]. In the presented study sward height during grazing period was 4.0–5.5 cm.

Using an indwelling pH measurement system enables the demonstration of circadian pH changes. It is in evidence that presented study cows were “off feed” for 6 hours on day 27 (Figure 1), which led to a distinct elevation of the reticuloruminal pH in all cows due to absent feed intake and increased salivation. Schwartzkopf-Genswein et al. [45] observed some animals with decreased feed intake, where limited production of fermentation acids led to an elevation of ruminal pH. 

There is strong evidence that the development of acidotic stages in the reticulorumen in cattle involves a strong interaction among feed intake, diet composition, feeding management, feeding behaviour, and the individual animal.

## 5. Conclusions

Reticuloruminal pH was significantly influenced by the diet. It can be concluded that SARA might occur under practical conditions in dairy cows during the period of feed changeover and when pasture feeding is combined with supplemental feeding of grain-based concentrates. The described indwelling pH measuring device is a very helpful tool and can also be used for practical purposes.

## Figures and Tables

**Table 1:** Mean reticuloruminal pH during feed changeover (phases 1–7), feed intake, and nutrient contents.

	Mean				Phases				*P*-value	SEM^1^	*R* ^²^
		Phase 1	Phase 2	Phase 3	Phase 4	Phase 5	Phase 6	Phase 7			
Mean pH value	6.43	6.58^a^	6.54^ab^	6.49^ab^	6.46^ab^	6.40^abc^	6.34^bc^	6.19^c^	0.006	0.15	68.98
SEM^1^	0.19	0.15^a^	0.17^a^	0.24^b^	0.21^ab^	0.19^ab^	0.18^ab^	0.19^ab^	0.502	0.07	29.95
24-h minimum pH	6.01	6.23	6.19	5.93	5.99	6.00	5.93	5.75	0.054	0.202	56.89
*t* < pH 6.3 (min/d)	491	258	321	417	448	436	675	884	0.053	308	56.89
*t* < pH 6.0 (min/d)	89	6^a^	6^a^	148^a^	71^ab^	55^ab^	68^ab^	261^b^	0.226	165.98	43.62
*t* < pH 5.8 (min/d)	28	0^a^	0^a^	53^b^	23^ab^	10^ab^	11^ab^	96^b^	0.688	97.6	29.89
*t* < pH 5.5 (min/d)	2	0^a^	0^a^	1^a^	5^a^	0^a^	0^a^	6^a^	0.848	8.28	23.74

Feed intake											
Hay (kg DM/d)	3.6	3.9	3.7	3.7	3.7	3.7	3.7	2.7	0	0.09	99.14
Grass Silage (kg DM/d)	3.7	7.8	3.6	4.1	3.1	3.7	3.6	0	0	1.60	81.74
Concentrate (g/kg DM)	1.8	2.8	1.8	1.8	1.8	1.8	1.8	1.3	0	0.08	98.26
Pasture (g/kg DM)	9.7	0	10.1	9.9	11.0	10.3	10.6	15.9	0	1.46	95.1
Daily DMI (kg DM)	18.8	14.4	19.2	19.5	19.6	19.5	19.6	19.9	0	0.77	94.75

Nutrient contents											
Crude protein (g/kg DM)	147	133	150	146	148	147	146	158	0	1.33	98.39
Fiber (g/kg DM)	211	240	210	212	206	209	208	188	0	7.42	91.96
Energy (MJ NEL/kg)	6.41	5.99	6.44	6.41	6.48	6.43	6.44	6.67	0	0.09	92.58
NDF (g/kg DM)	414	454	405	415	407	413	416	391	0.130	8.55	93.08
ADF (g/kg DM)	234	275	231	235	227	232	231	208	0	10.12	91.31
ADL (g/kg DM)	30	34	29	30	29	30	30	27	0	0.72	95.98

^a–c^Least squares means within a row without a common superscript differ significantly (*P* < 0.05).

^1^SE of least squares means.

**Table 2:** Effects of different dietary treatments (GC, GH, and G) on reticuloruminal pH, standard deviation, mean of daily minimum (nadir), and the time (min/d) pH were below 6.3, 6.0, 5.8, and 5.5.

	Mean	Treatments			Statistics		
		1	2	3			
		GC	GH	G	*P* value	SEM^1^	*R* ^²^
Mean pH value	6.31	6.01^a^	6.56^b^	6.36^b^	0.0001	0.18	0.54
SE	0.17	0.20^a^	0.15^b^	0.16^b^	0.0241	0.036	0.3
24-h minimum pH	5.91	5.58^a^	6.21^b^	5.95^b^	0.0001	0.2	0.58
*t* < pH 6.3 (min/d)	602	1126^a^	97^b^	583^c^	0.001	323.99	0.6
*t* < pH 6.0 (min/d)	241	621^a^	12^b^	91^b^	0.0003	241.75	0.54
*t* < pH 5.8 (min/d)	125	347^a^	0^b^	26^b^	0.0051	188.83	0.4
*t* < pH 5.5 (min/d)	35	101^a^	0^b^	3^b^	0.0021	51.3	0.44

Feed intake							
Hay (kg DM/d)	2.0	1.0	1.0	4.0	0	0.61	0.76
Concentrate (kg DM/d)	2.0	4	1.75	0	0	0.66	0.88
Pasture (kg DM/d)	16.3	15.5	16.7	16.8	0.0432	0.86	0.34
Daily DMI (kg DM/d)	20.2	20.7	19.7	20.1	0.0033	0.45	0.53

Nutrient values							
Crude protein (g/kg DM)	168	163	168	172	0	1.94	0.8
Fiber (g/kg DM)	183	165	180	204	0	4.85	0.93
Energy (MJ NEL/kg)	6.68	6.83	6.73	6.48	0	0.06	0.89
NDF (g/kg DM)	385	359	379	416	0	7.16	0.93
ADF (g/kg DM)	206	184	201	234	0	6.22	0.93
ADL (g/kg DM)	27	24	26	29	0	0.66	0.91

^a–c^Least squares means within a row without a common superscript differ significantly (*P* < 0.05).

^1^SE of least squares means.
